# Placenta accreta spectrum in the 21st century: Challenging dogma and redefining disorder

**DOI:** 10.1371/journal.pmed.1005142

**Published:** 2026-06-12

**Authors:** Eric Jauniaux, Helena C. Bartels, Yalda Afshar

**Affiliations:** 1 EGA Institute for Women’s Health, Faculty of Population Health Sciences, University College London (UCL), London, United Kingdom; 2 Maternal-Fetal Medicine Division, Department of Obstetrics and Gynaecology, Mount Sinai Hospital, Toronto, Canada; 3 Division of Maternal Fetal Medicine, Department of Obstetrics and Gynecology, David Geffen School of Medicine, University of California, Los Angeles, Los Angeles, California, United States of America; 4 Molecular Biology Institute, School of Medicine, University of California, Los Angeles, California, United States of America

## Abstract

*In this Perspective, Jonathan Evans and colleagues discuss why restricting access to joint replacement surgery based on BMI alone is not supported by evidence, and highlight how such rest* r*ictions risk exacerbating stigma, inequity and avoidable harm to those who would benefit from surgery*.

## Teaser

Placenta accreta spectrum (PAS) is a serious pregnancy complication caused by abnormal placental attachment to the uterus. In this Perspective, Eric Jauniaux and colleagues discuss emerging evidence that challenges our long-held pathophysiological understanding of PAS, and argue that a critical reassessment of definition, diagnosis, and management is overdue.

**Fig 1:**
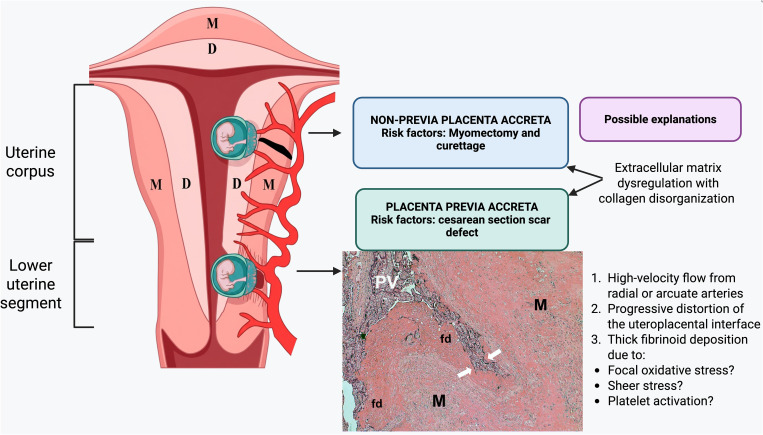
Accreta placentation in different parts of the uterine wall and proposed mechanisms. Placentation in the uterine corpus above a well-healed scar can be associated with abnormal superficial adherence of the placenta that may not be detectable on prenatal imaging. Placentation inside a cesarean scar defect with major remodeling of the myometrium (M) of the lower uterine segment can lead to abnormally attached placental villi (PV) deep within a scar gap (increta). Possible explanations for PAS include extracellular matrix dysregulation with collagen disorganization, high-velocity uteroplacental blood flow on ultrasound imaging, progressive anatomical distortion of the uteroplacental interface, and thick fibrinoid deposition (fd) between the normal PV and scarred myometrium. Created in BioRender. Bartels, **H.** (2026) https://BioRender.com/wrnhin6.

Placenta accreta spectrum (PAS) is a disorder of placentation characterized by the abnormal attachment of part of the placenta villous tissue to the uterine myometrium (see Fig 1). When unrecognized at birth, attempts at manual placental removal can precipitate massive hemorrhage and significant maternal morbidity and mortality. First described by Irving and Hertig in 1937 [1], PAS was defined clinically as abnormal placental adherence at delivery and histologically by the absence of an intervening decidual layer; criteria that remain the diagnostic standard today. However, emerging evidence challenges the long-held pathophysiological paradigm of primary placental invasion, instead implicating disordered placentation secondary to uterine post-surgical remodeling.

## Revisiting the pathophysiology of placenta accreta spectrum (PAS)

The severity of PAS has been graded as: superficial villous adherence to the myometrium (placenta creta/vera); villous tissue invading deep inside the myometrium (placenta increta); and villous tissue crossing the entire uterine wall thickness and beyond (placenta percreta) (see Fig 1) [2]. The assumption that morphologically normal placental villous tissue can either simply adhere to the uterine wall or cross it to invade other pelvic organs has been challenged [3]. Firstly, the decidua—an essential layer in the first trimester of pregnancy resulting from the transformation of the uterine endometrium—becomes thinner as pregnancy advances and is discontinuous or absent over most of the placental-myometrium interface in the third trimester [4]. Seconddly most samples from hysterectomies performed in the management of placenta previa accreta (attachment in a previous low uterine segment cesarean scar) show thick fibrinoid deposition at the uteroplacental interface, with secondary distortion of the Nitabuch membrane, the layer where the placenta normally detaches from the uterus at delivery (Fig 1). Together, these findings indicate that there is more than a simple absence of the decidua to explain the abnormal attachment of part of the placenta in accreta placentation.

There is no evidence that normal villous tissue can cross the entire uterine wall [3]. Even in severe cases associated with placenta previa and lower uterine segment dehiscence, placental tissue remains contained within a thin scar shell [3]. Apparent extrauterine placental extension is the result of surgical manipulation and dissection or uterine rupture rather than true biological invasion. It has, in the past, led to a false diagnosis of placenta percreta [2,3]. Meanwhile, differential gene expression in the placental basal plate between accreta and nonaccreta areas has revealed that, across all cell types, endothelial-stromal cell populations exhibit the greatest differences in gene expression, driven by changes in collagen, growth factor, and angiogenesis-related genes [5]. These transcriptional and protein changes in the stroma of PAS have shifted the etiologic explanation away from “invasive trophoblast” toward “loss of boundary limits”, with uncontrolled migration of extravillous placental cells rather than excessive villous invasion [3,5]. These new findings have challenged the clinical concept that all grades of PAS result from abnormal placental invasion of the uterine wall, which is like a cancer, requires a cesarean hysterectomy as the primary management strategy [2,3].

## Cesareans and shifting epidemiology of PAS

Modern low-segment cesarean section increases the risk of both placentation in the lower uterine segment (low-lying placenta and placenta previa) and placenta previa accreta in subsequent pregnancies [6]. Cesarean rates have tripled in most middle- and high-resource countries since the 1980s, changing the epidemiology of PAS [7]. Currently, over 90% of patients with a diagnosis of PAS at birth present with a low-lying placenta or placenta previa [2,3]. A systematic review of the prevalence of PAS in obstetric populations from 1982 to 2018 reported a pooled prevalence of 0.17% among livebirths, with substantial heterogeneity across studies [8]. However, fertility rates have fallen below 1.5 in high-resource countries, suggesting that the prevalence of PAS will remain stable or decline in most Western countries. By contrast, PAS is becoming a major healthcare issue in urban populations in middle- and low-resource countries with fertility rates > 2 and cesarean section rates > 40% [7]. The shifting epidemiology underscores the growing global relevance of accurately defining and managing PAS.

## Towards improved diagnosis and management of PAS

The lower uterine segment is much thinner and contains fewer myofibres than the upper segment (uterine corpus) and is more prone to the development of permanent scar defects (Fig 1). The development of part of the placenta inside a cesarean scar defect brings the corresponding villous tissue abnormally close to the large uterine arteries (radial or arcuate) of the uterine periphery [2]. Patients with a history of multiple cesarean births and a placenta previa accreta often present on ultrasound imaging with extended remodeling of the uterine wall and major changes in the uteroplacental and intraplacental circulations [2]. These changes are detectable antenatally, which is pivotal for their management, as they require delivery by an expert multidisciplinary team capable of performing complex cesarean sections, including cesarean hysterectomies [2,9].

Standard gynecologic surgical procedures, such as uterine curettage (removal of tissue from the lining of the uterus), have been associated with the development of nonprevia PAS in subsequent pregnancies [2,3]. A recent study on the role of collagen has found disorganized fibrillar collagen deposition with disrupted border-integrity architecture in human placenta accreta, and matrix disorganization at the site of abnormal placental adherence in a surgical mouse model [10]. This could explain the abnormal adherence of part of the placenta in superficial scars of the uterine corpus and contributes to the mechanisms leading to PAS in the lower uterine segment. However, as placentation in a superficial scar of the uterine corpus is not associated with striking modifications in the uterine wall structure or vasculature on ultrasound, nonprevia PAS are difficult to identify antenatally [2]. Within this context, even when only diagnosed at birth, their location in the thick wall of the uterine corpus should allow for conservative surgical management with uterine preservation, such as partial myometrial resection of the accreta lesion [2,3].

In the last decade, an increasing number of observational studies on PAS have included patients presenting with a difficult piecemeal removal of the placenta, absence of spontaneous placental separation 30 min after birth despite active management, and retained placental fragments on ultrasound requiring curettage after vaginal birth [3]. These clinical descriptions were previously used in general obstetrics to report placental retention and/or uterine atony, and all refer to complications of placental delivery involving the uterine corpus. These criteria are inherently subjective and risk conflating true pathological placental attachment with nonaccreta variations in placental separation. Diagnostic ambiguities have contributed to the overdiagnosis and overtreatment of superficial grades of PAS (placenta creta/vera) with peripartum hysterectomy as the primary management strategy [8,11]. Of particular concern is the impact of such management in primiparous patients with no uterine surgical history and the long-lasting impact of a major surgical procedure on their physical and emotional health, fertility, and their relationship with their partner [12].

Our understanding of PAS has evolved. PAS is not a primary anomaly of placental development but the consequence of implantation and placentation in a part of the uterine wall that has been permanently damaged by surgery, the impact of which is far greater in the physiologically thinner lower segment than the thicker upper segment. There is therefore an urgent need to align PAS management with modern evidence-based clinical data and our current understanding of the underlying biology.
